# PASSPORT-seq: A Novel High-Throughput Bioassay to Functionally Test Polymorphisms in Micro-RNA Target Sites

**DOI:** 10.3389/fgene.2018.00219

**Published:** 2018-06-15

**Authors:** Joseph Ipe, Kimberly S. Collins, Yangyang Hao, Hongyu Gao, Puja Bhatia, Andrea Gaedigk, Yunlong Liu, Todd C. Skaar

**Affiliations:** ^1^Division of Clinical Pharmacology, Department of Medicine, Indiana University School of Medicine, Indianapolis, IN, United States; ^2^Department of Pharmacology and Toxicology, Indiana University School of Medicine, Indianapolis, IN, United States; ^3^Department of Medical and Molecular Genetics, Indiana University School of Medicine, Indianapolis, IN, United States; ^4^Center for Computational Biology and Bioinformatics, Indiana University School of Medicine, Indianapolis, IN, United States; ^5^Division of Clinical Pharmacology, Toxicology and Therapeutic Innovation, Children’s Mercy Kansas City, Kansas City, MO, United States

**Keywords:** SNP, functional testing, genetic variants, miRNA, high-throughput screening assays, 3′ UTR

## Abstract

Next-generation sequencing (NGS) studies have identified large numbers of genetic variants that are predicted to alter miRNA–mRNA interactions. We developed a novel high-throughput bioassay, PASSPORT-seq, that can functionally test in parallel 100s of these variants in miRNA binding sites (mirSNPs). The results are highly reproducible across both technical and biological replicates. The utility of the bioassay was demonstrated by testing 100 mirSNPs in HEK293, HepG2, and HeLa cells. The results of several of the variants were validated in all three cell lines using traditional individual luciferase assays. Fifty-five mirSNPs were functional in at least one of three cell lines (FDR ≤ 0.05); 11, 36, and 27 of them were functional in HEK293, HepG2, and HeLa cells, respectively. Only four of the variants were functional in all three cell lines, which demonstrates the cell-type specific effects of mirSNPs and the importance of testing the mirSNPs in multiple cell lines. Using PASSPORT-seq, we functionally tested 111 variants in the 3′ UTR of 17 pharmacogenes that are predicted to alter miRNA regulation. Thirty-three of the variants tested were functional in at least one cell line.

## Introduction

Large scale sequencing studies and genome-wide association studies (GWASs) have identified 1000s of genotype–phenotype associations (Welter et al., 2014). Some of the phenotype-associated variants alter gene function and many of them are in linkage disequilibrium with the functional variants. The functional impacts of variants can be predicted using bioinformatic algorithms, but the *in silico* predictions are often incorrect and need experimental validation. While there are several experimental methods to functionally test variants, most do not have the capacity to simultaneously test the large number of variants.

Nearly 90% of genetic variants associated with phenotypes have been described to be located in non-coding regions such as the untranslated regions (UTRs) (Hindorff et al., 2009). Variants, including single nucleotide polymorphisms (SNPs) within non-coding regions, can impact gene expression in several ways; one example is by altering the interaction between mRNAs and micro-RNAs (miRNAs). Polymorphisms within miRNA-binding sites have been implicated in diseases such as cancer (Pelletier and Weidhaas, 2010; Iuliano et al., 2013), Alzheimer’s disease (Liu et al., 2017), and diabetes (Elek et al., 2015).

miRNAs are 21–23 nucleotide long RNAs that post-transcriptionally silence genes or reduce their expression levels by complementarily binding to target sites within mRNAs. More than 29,000 human mRNAs are collectively targeted by are over 2500 miRNAs (Kozomara and Griffiths-Jones, 2014). Several different miRNA binding sites may be present on one mRNA and many contain genetic variations. To date, over 400,000 SNPs have been identified in miRNA binding sites (Liu et al., 2012). Interestingly, only about 32,000 have a minor allele frequency greater than 1% classifying most of them as rare variants (Liu et al., 2012). Thus, tests to identify functional SNPs affecting miRNA binding, here referred to as mirSNPs, will involve screening a large number of variants.

Testing these large number of mirSNPs using GWAS requires statistical correction for multiple testing, such as the Bonferroni correction. The low minor allele frequency of many causal variants, and routine multiple comparisons corrections make it very difficult, or impossible, to statistically identify functionally relevant variants in genome-wide studies. Consequently, in GWAS, impractically large numbers of subjects from diverse populations would be required to identify rare functional variants that are statistically significant. Lowering the statistical threshold or not correcting for multiple comparisons increases the sensitivity to detect rare variant associations, but results in the detection of many false positives signals. Despite some technical challenges, high-throughput *in vitro* approaches have been implemented that are specific to variants in certain non-coding regions, such as splice-junctions (Soemedi et al., 2014) and promoters (Kwasnieski et al., 2012; Melnikov et al., 2012). However, we are not aware of any high-throughput assays available to functionally test variants in miRNA binding sites (Ipe et al., 2017).

We developed PASSPORT-seq (parallel assessment of polymorphisms in miRNA target-sites by sequencing), a high-throughput bioassay that involves pooled synthesis, parallel cloning and single-well transfection followed by next-generation sequencing (NGS) to functionally test 100s of mirSNPs at once. This assay produced results that are reproducible and consistent with luciferase reporter assays, a gold-standard platform widely used to assess gene expression *in vitro*. We also demonstrate the application of this assay to test 111 genetic variants that are predicted to alter miRNA regulation of 17 pharmacogenes.

## Materials and Methods

### Selection of mirSNPs

RNA samples from thirty human livers were sequenced using SOLiD^®^ technology (Thermo Fisher Scientific, Waltham, MA, United States). SNPs in the 3′ UTRs were identified (O’Leary et al., 2016) and an 8-base pair region on either side of the reference and variant alleles was analyzed using TargetScan (Lewis et al., 2005) to identify SNPs that were in miRNA seed binding regions. SNPs that altered the predicted miRNA seed binding sites were considered for further analysis. For assay development, 84 SNPs that were associated with allele-specific expression in the sequencing dataset were selected. A flowchart representing the selection process of the 84 test mirSNPs is shown in Supplementary Figure 1. In addition, we selected 16 mirSNPs from the SomamiR database (Bhattacharya et al., 2013) that have been linked with cancer. The list of 100 SNPs used for assay development are listed in Supplementary Table 1. Similarly, 111 mirSNPs located in the 3′ UTR regions of 17 pharmacogenes- the core absorption, distribution, metabolism, and excretion (ADME) genes^1^, PXR, CAR, and HNF4α which showed allele specific expression in the sequencing dataset were selected to demonstrate the application of the assay. The list of these 111 SNPs are listed in Supplementary Table 4. The RNA analysis and genotyping was approved by the Indiana University Institutional Review Board.

### Test Sequence Design

The 5′ and 3′ flanking regions for each SNP were obtained from dbSNP. A 32-nucleotide region which contained either the variant or reference nucleotide flanked by nine nucleotides on the 3′ end and 22 nucleotides on the 5′ end was selected as the test sequence. Two-hundred such regions (100 reference and 100 variant) were selected to test 100 SNPs. Universal primer binding regions were added on the 5′ (GTAATTCTAGGAGCTC) and 3′ (CGTTCTAGAGTCGGG) end of each test region. The final test fragment was 63 nucleotides in length (see Supplementary Figure 2). The 200 test fragments were commercially synthesized as pooled single-stranded DNA oligonucleotides (Oligomix^®^, LC Sciences, Houston, TX, United States). The pool contained 10–50 attomoles of each sequence. The oligonucleotides were synthesized as single-stranded DNA and was diluted 1:5. One μL of the diluted Oligomix^®^ was amplified in a 50 μL PCR reaction using 0.3 μM universal primers and 25 μL 2X CloneAmp^TM^ HiFi PCR premix (Takara, Mountain View, CA, United States). PCR conditions used were: 98°C (10 s), 53°C (5 s), and 72°C (5 s) for 35 cycles.

Seven SNPs were also tested in individual luciferase assays using 63-nucleotide long single stranded oligonucleotides that were individually synthesized (reference and variant); (Integrated DNA Technologies, Coralville, IA, United States), made double stranded as described for pooled oligonucleotides, and cloned into the pIS-0 plasmid.

### Plasmid Library Preparation

The pIS-0 vector (plasmid 12178; Addgene, Cambridge, MA, United States) (Yekta et al., 2004) (see Supplementary Figure 3) was linearized with *Sac*I-HF^®^ and *Bmt*I-HF^®^ restriction endonucleases (New England Biolabs, Ipswich, MA, United States) and purified using QIAquick^®^ PCR spin columns (Qiagen, Germantown, MD, United States). Plasmid assembly was performed using 40 ng of linearized plasmid and 2 μL of unpurified PCR product containing double-stranded test oligonucleotides using the NEBuilder^®^ HiFi DNA assembly kit (NEB, Ipswich, MA, United States) per manufacturer’s instructions. The universal primers used to amplify the test-oligonucleotide pool also served as the flanking homology regions for the NEBuilder^®^ assembly. Two μL of the NEBuilder^®^ assembly product were transformed into 60 μL chemically competent *E. coli* (transformation efficiency > 5 × 10^8^ cfu/μg) (Takara, Mountain View, CA, United States) and plated on six standard 100 mm LB-agar plates containing 100 μg/ml ampicillin. After overnight incubation, all colonies were dislodged from the plates by adding 2 ml LB-broth containing 100 μg/ml ampicillin and agitation using 10–20 ColiRollers^TM^ glass beads (EMD Millipore, Billerica, MA, United States). The colonies harvested from the six plates in LB-broth were pooled together. The liquid culture was incubated at 37°C for 5 h after which plasmids were isolated using 10 QIAprep^®^ Spin miniprep columns (Qiagen, Germantown, MD, United States) as per manufacturer’s instructions. Column elutions were combined to create the plasmid library that was used for downstream experiments. The plasmid DNA concentration was determined using a Quant-iT^TM^ DNA Broad Range kit (Thermo Fisher Scientific, Waltham, MA, United States).

### Sanger Sequencing

To determine the representation of the test constructs in the plasmid library, 28 individual colonies were grown in LB-broth containing 100 μg/ml ampicillin. Plasmids were isolated using QIAprep^®^ Spin miniprep columns (Qiagen, Germantown, MD, United States) as per manufacturer’s instructions and Sanger sequenced using a primer (GTGGTTTGTCCAAACTCATC) near the test insert (ACGT, Inc., Wheeling, IL, United States).

### Transfection of Cells in Culture

The plasmid library was used to transfect three different human cell lines: HEK293 (embryonic kidney), HepG2 (liver carcinoma), and HeLa (cervical cancer). Cells were seeded at a density of 0.9 × 10^5^ cells per well into 24- well plates. The cells were transfected 24 h after plating with 500 ng/well of the pIS-0 plasmids. Ten ng of pGL4.74, a Renilla luciferase reporter plasmid, was added to each well as a transfection control. Transfection was performed using 50 μL transfection-mix in Opti-MEM^®^ (Life Technologies, Carlsbad, CA, United States) containing 1.5 μL Lipofectamine^®^ 3000 (Life Technologies, Carlsbad, CA, United States) per the manufacturer’s instructions. Opti-MEM^®^ and culture media were used with no antibiotics.

### RNA Isolation and cDNA Synthesis

Transfected cells were incubated for 48 h, lysed *in situ* and total RNA isolated using a RNeasy^®^ purification kit with the optional DNase treatment (Qiagen, Germantown, MD, United States). RNA was quantified using the Quant-iT^TM^ RNA Broad Range kit (Thermo Fisher Scientific, Waltham, MA, United States) and cDNA synthesized from 800 ng of total RNA using the QuantiTect^®^ Reverse Transcription kit (Qiagen, Germantown, MD, United States).

### Molecular Barcoding

**FIGURE 1:**
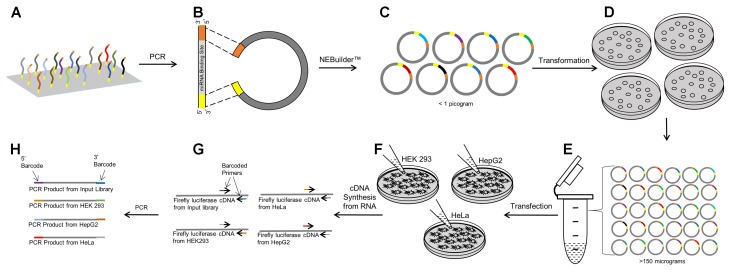
Workflow of the PASSPORT-seq bioassay. **(A)** 100 Reference and variant miRNA binding regions each with the same 15–20 bp flanking sequence was synthesized as an oligonucleotide pool. **(B)** Using the flanking universal sequences, the oligonucleotide pool was amplified and made double stranded by PCR. pIS-0 plasmid was linearized by restriction enzymes. **(C)** The double stranded oligonucleotides were inserted into the linear plasmid using the NEBuilder^TM^ gene assembly system. **(D)** Chemically competent bacteria were transformed with the plasmid pool containing the test miRNA binding regions. Transformed bacteria were plated on four plates. **(E)** All colonies from the plates were harvested, combined and scaled up in liquid culture. Plasmids were isolated from the liquid culture. **(F)** Three cell lines were transfected with the plasmid pool and incubated for 48 h after which cDNA was prepared from total RNA. **(G)** miRNA binding regions were amplified using universal primers that were uniquely barcoded for replicates within cell lines and for the input plasmid pool. **(H)** The barcoded PCR products were combined to form the sequencing pool.

Using the cDNAs from the transfected cells, the miRNA binding sites within the 3′ UTR of the luciferase genes were amplified in 50 μL PCR reactions using 0.3 μM flanking universal primer sand 25 μL 2X CloneAmp^TM^ HiFi PCR premix (Takara, Mountain View, CA, United States). In a separate reaction for each sample, 2 μL of cDNA and 1 pg of the input plasmid pool was used as PCR template. PCR conditions used were: 98°C (10 s), 54°C (5 s), and 72°C (5 s) for 25 cycles. A 6-nucleotide unique molecular barcode was added to the 5′-end of both the forward and reverse primer (see Supplementary Table 2). The input pools (*n* = 4 replicates) and the five biological replicates in the three different cell lines were each ‘barcoded’ by a unique pair of sequences. The barcoded PCR products were purified using a MinElute^®^ PCR Purification kit (Qiagen, Germantown, MD, United States). The barcoded libraries were combined in equimolar concentrations to create a sequencing pool with 19 different molecular barcodes. A schematic representation of the steps involved in creating this sequencing pool is shown in **Figure 1**.

### Next-Generation Sequencing

The pooled PCR products were sequenced using a modified protocol for the Ion Proton^TM^ system (Thermo Fisher Scientific, Waltham, MA, United States). Briefly, the sequencing library was created by end-polishing the barcoded PCR products, followed by adapter ligation and amplification. The resulting library was quantified and its quality accessed with the Agilent Bioanalyzer (Agilent Technologies, Santa Clara, CA, United States). Eight microliters of the 100 pM library were then applied to Ion Sphere Particles to prepare the sequencing template. The template was amplified using Ion OneTouch 2. The Ion Sphere Particles with the amplified template were loaded onto an IonPI^®^ chip and sequenced on the Ion Proton system per manufacturer’s instructions. Approximately, 41 million reads were generated from each sequencing run. Raw reads were generated as fastq files for bioinformatic analysis. Sequencing data has been made publicly available through GEO (Accession No. GSE111845).

### Bioinformatic Analysis

The raw reads were aligned to the reference library containing the 200 test sequences (TMAP- Ion Torrent Suite^®^, Thermo Fisher Scientific, Waltham, MA, United States). The reads that aligned to the reference library were filtered to retain reads with a mapping quality greater than 20 and further filtered to include only those sequences with perfect barcodes at both ends.

Differential expression analysis compared the expression of each variant to its respective reference allele for all SNPs. To account for differences in the concentrations of the variant and reference plasmids that were used for the transfections, a plasmid input correction factor for each target site was calculated as the average of the number of reads from the variant plasmid divided by the number of reads from the reference plasmid across four replicates of the plasmids. The reads from the variant alleles for all biological replicates were divided by the input correction factor. The corrected read counts were fit into a generalized linear model using EdgeR (Robinson et al., 2010) assuming a negative binomial distribution. Biological replicates and the genotype were used as covariates. *p*-Values and log_2_ fold-change of the variant alleles compared to the respective reference alleles were derived using a likelihood ratio test on the genotype variable in the generalized linear model. The *p*-values were corrected for a false discovery rate (FDR) using the Benjamini and Hochberg algorithm (Benjamini and Hochberg, 1995). The EdgeR script can be found in Supplementary File 1.

The two sequencing runs, each with five biological replicates, were analyzed together by fitting the number of reads for 10 pairs of variant and reference alleles (five from each experiment). The different sequencing runs were included as an additional covariate. The statistical analysis was performed as described above.

### Luciferase Reporter Assay

Genetic variants, including mirSNPs have been functionally tested using a reporter plasmid such as the pIS-0 vector (Yekta et al., 2004; Adams et al., 2007; Ramamoorthy et al., 2012). This plasmid contains the firefly luciferase gene whose expression can be quantified either by qPCR or by the luciferase reporter assay. The reference or variant allele version of the predicted miRNA binding sites were cloned into the 3′ UTR of the luciferase gene within the plasmid. The plasmids were then transfected into cells as described above. Forty-eight hours after transfection, cells were lysed *in situ* and Dual-Luciferase^®^ assays were performed per manufacturer’s instructions (Promega, Madison, WI, United States). The luciferase reporter activity was measured using a 96-well plate-reader (BioTek, Winooski, VT, United States). The firefly luciferase activity was normalized to that of Renilla luciferase in each well. The ratio of the normalized luciferase activity from the variant and reference plasmid provides a relative measure of SNP-mediated differential mRNA expression.

## Results

The traditional luciferase reporter assay is useful in low-throughput experiments, but is not a practical and cost-effective method to test the 1000s of mirSNPs identified at a genome-wide scale. As a novel approach, we modified the luciferase reporter assay to develop PASSPORT-seq that can functionally test 100s of mirSNPs in parallel. Since one of the mechanisms of miRNA regulation is by degrading mRNA, this assay was specifically designed to evaluate the impact of genetic variation in miRNA binding sites on mRNA expression. We recognize that miRNAs also alter mRNA translation, however measuring protein levels does not distinguish between the impact on mRNA vs. translation and thus would not provide the same mechanistic insights. We identified 100 variants in predicted seed sequences of miRNA binding sites, and cloned the binding sites into the pIS-0 luciferase plasmid; each contained either the reference or variant nucleotide of 100 selected mirSNPs. The pool of the resulting 200 plasmids was then transfected into three cell lines and the luciferase gene expression measured by NGS. A difference in mRNA expression between the reference and the variant plasmids indicated a functional mirSNP.

### Cloning Efficiency and Plasmid Representation

To test the efficiency of the parallel cloning, plasmids from 28 individual colonies were isolated and the sequence of the inserts were determined by Sanger sequencing. Out of the 28 colonies, 25 contained inserts without errors; of those, 24 were unique sequences suggesting that the cloning efficiency was high and there was negligible sequence-bias in the plasmid pool. Furthermore, as described below, all 200 sequences were observed in the NGS of the entire pool.

### Reproducibility Across Biological Replicates

To test the reproducibility of the PASSPORT-seq bioassay, we first performed the assay with five biological replicates in each of the three cell lines and compared the number of reads from two of the five biological replicates. The input plasmid libraries had representation of all 100 allelic pairs. The R^2^ value for the comparison of sets of two of the five input normalized-biological replicates within the same sequencing run was between 0.68 and 0.98 (*p* < 0.05; Supplementary Figure 4) demonstrating highly reproducible results within a run.

### Reproducibility Across Runs

**FIGURE 2:**
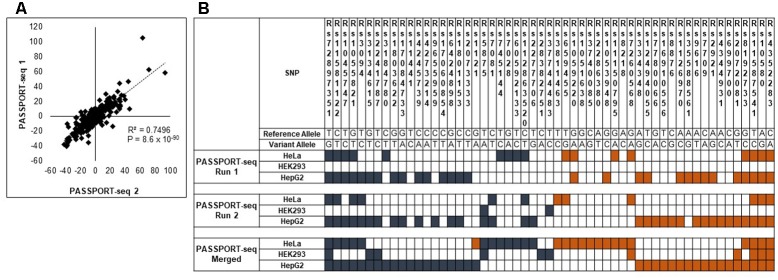
Validation of the PASSPORT-seq assay. **(A)** Correlation between the percent-change of variant alleles compared to respective reference alleles observed in the experimental and validation PASSPORT-seq runs. Each run contained five biological replicates tested in three cell lines. The graph includes combined average data from all three cell lines. **(B)** Functional mirSNPs identified by the PASSPORT-seq assay. For each SNP, the observed percent change in the expression of the variant allele compared to the respective reference allele in predicted miRNA binding site was calculated Statistically significant changes after correction for multiple testing using Benjamini and Hochberg algorithm are indicated by colored boxes. Blue boxes indicate a reduction in the variant allele expression and Orange boxes indicate increased expression. Results from the experimental and validation runs are shown. Additionally, results of the analysis with merged data from experimental and validation runs are also represented.

Next, we repeated the PASSPORT-seq assay again with another five biological replicates in each of the three cell lines to validate the observed results. A separate cDNA and sequencing library was created for the experiments. A strong correlation (*R*^2^ = 0.98; *p* < 0.05) was observed in the results from the plasmid pool from the first sequencing run and those from the second sequencing run (see Supplementary Figure 5). There was a high correlation (*R*^2^ = 0.74; *p* < 0.05) in the results between the first and second set of biological replicates (**Figure 2** and Supplementary Figure 6). This strong correlation between results of the first sequencing run with those of the second sequencing run across the three cell lines demonstrates the high reproducibility of the observed results.

### Identification of Functional mirSNPs

**FIGURE 3:**
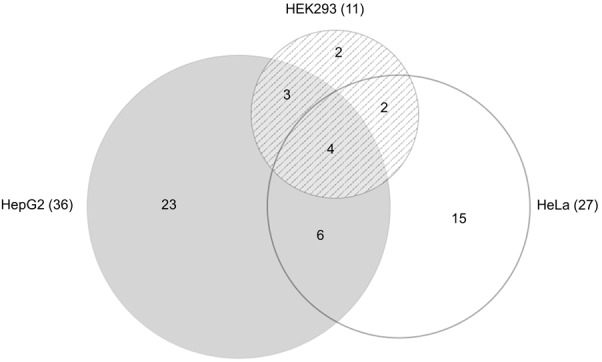
Functional SNPs in the cell lines. Venn diagram (Micallef and Rodgers, 2014) showing the number of unique and overlapping functional SNPs that were identified by the PASSPORT-seq assay in the three cell lines. Total number of functional SNPs identified in each cell line is indicated in parenthesis.

Of the 100 mirSNPs tested, 69 showed statistically significant (*p* < 0.05) differences in expression between the variant and its respective reference allele in at least one cell line (**Figure 2B**). In HEK293, HepG2, and HeLa a significant effect was seen in 27, 44, and 39 mirSNPs, respectively (see Supplementary Figure 7). Due to the large number mirSNPs tested, the results were corrected using the Benjamini and Hochberg procedure across cell lines. This conservative threshold (FDR ≤ 0.05) was met by 55 mirSNPs in at least one cell line with 11, 36, and 27 in HEK293, HepG2, and HeLa cells, respectively. Because these variants were informatically predicted to be functional, this may be an overly conservative statistical correction. The effect of the mirSNPs was cell line- specific; four SNPs were functional across all three cell lines, while others were functional in either two cell lines or unique to one cell line (**Figure 3**).

### Validation With Traditional Luciferase Assays

**FIGURE 4:**
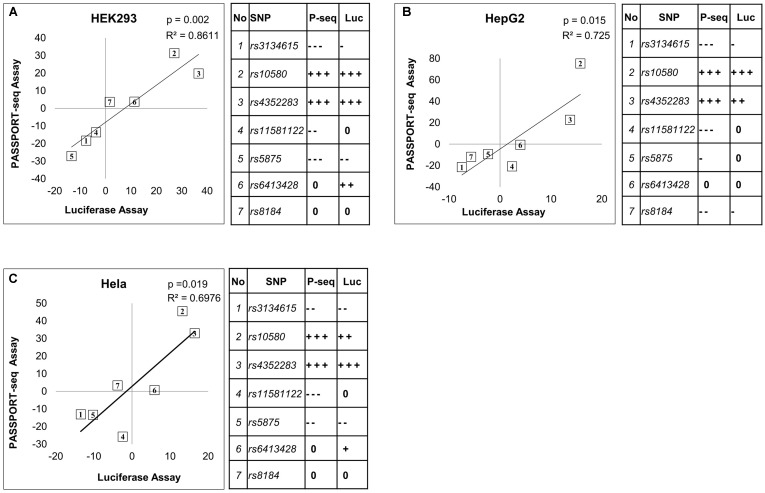
Comparison of the results from the PASSPORT-seq assay with those from the luciferase reporter assay. Correlation between results of the PASSPORT-seq assay (P-seq) and the luciferase reporter assay (Luc); in **(A)** HEK293, **(B)** HepG2 cells, and **(C)** HeLa cells. The results of the two assays are represented as the percent-change observed in the variant allele compared to its respective reference allele. Tables show the magnitude of change observed in each sample using the two different assays: < 5% = 0, ≥ 5 to < 10% = –/+, ≥ 10 to < 15% = --/++, ≥ 15% = --/+++. ‘–’ indicate decreased expression and ‘+’ indicate increased expression. The rs numbers of the SNPs can be identified based on the table.

Twenty-one of the results were validated using traditional individual luciferase transfection experiments. The variant and reference allele binding sites of the selected mirSNPs were individually cloned into the 3′ UTR of the luciferase gene within the pIS-0 plasmid and transfected into HEK293, HepG2, and HeLa cells. This included seven of the miRNA target sequences, each with reference and variant sequences, tested in three cell lines for a total of 21 validations. Within each cell line, the luciferase activity in the cells transfected with the reference plasmid was compared to the activity in the cells transfected with the variant plasmids. The effect of the variants in these individual luciferase assays were compared with the results from the PASSPORT-seq assay (**Figure 4**). In 17 of the 21 comparisons, the statistical significance of the results and the direction of the effect of the variant matched the PASSPORT-seq results (Supplementary Figure 8 and Supplementary Table 3). In an additional two comparisons (rs3134615 in HeLa and HEK293 cells), the results were statistically significant in one assay, but not the other, but the direction and magnitude of effect of the variant in the PASSPORT-seq was similar to the luciferase assay in both cell lines (Supplementary Figure 8). Thus, the results were very similar in 19 of the 21 comparisons (>90%).

### Application of PASSPORT-seq

**FIGURE 5:**
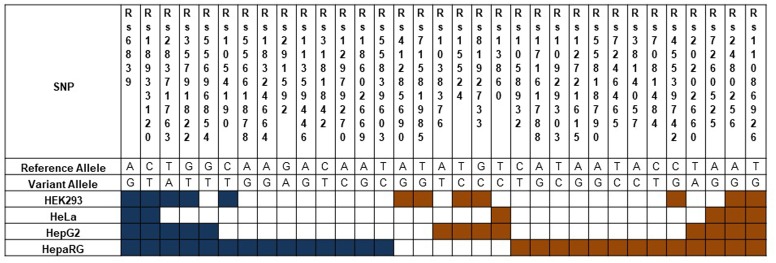
Functional mirSNPs in pharmacogenes identified by the PASSPORT-seq assay. For each SNP, the observed percent change in the expression of the variant allele compared to the respective reference allele in predicted miRNA binding site was calculated. Statistically significant changes after correction for multiple testing using Benjamini and Hochberg algorithm are indicated by colored boxes. Blue boxes indicate a reduction in the variant allele expression and Orange boxes indicate increased expression.

**FIGURE 6:**
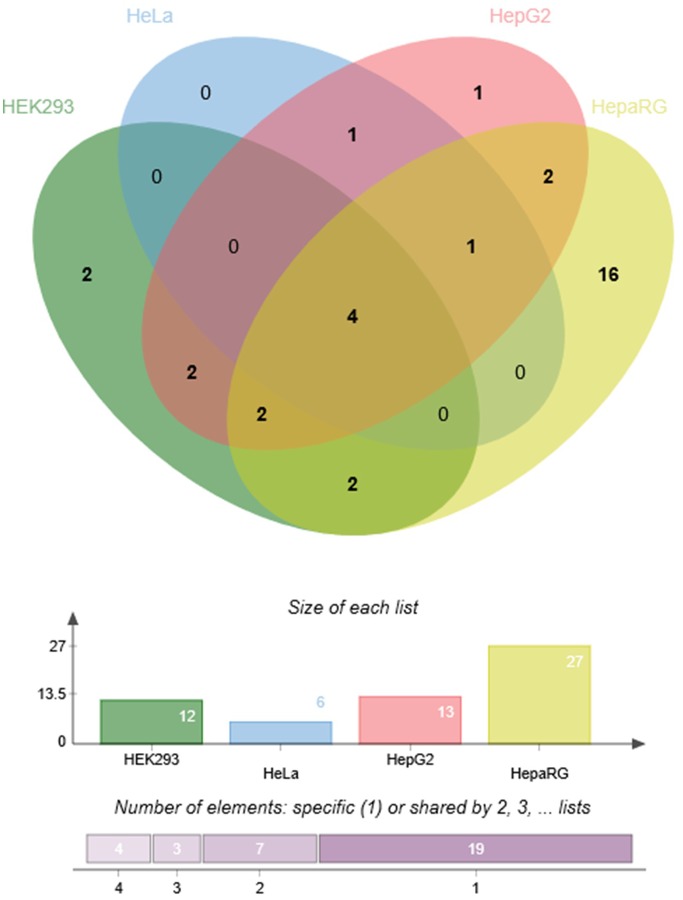
Cell line-specificity of functional mirSNPs in pharmacogenes. Venn diagram (Bardou et al., 2014) showing the number of unique and overlapping functional mirSNPs in the four cell lines tested.

To demonstrate the utility of this assay, mirSNPs predicted to alter miRNA regulation of 17 pharmacogenes were selected for functional testing from the RNA-seq dataset described earlier. These variants were functionally tested using the PASSPORT-seq assay in HeLa, HepG2, HEK293, and HepaRG (hepatic cells that retain characteristics of primary human hepatocytes) cells. Out of the 111 genetic variants tested, the effect of 33 variants were statistically significant in at least one cell line, including 6, 13, 12, and 27 in HeLa, HepG2, HEK293, and HepaRG cells, respectively (**Figure 5** and Supplementary Table 4). The effects of several mirSNPs were shown to be cell line-specific. Only four mirSNPs had significant effects in all the four cell lines (**Figure 6**). The effect of a genetic variant (rs12979270), located in the 3′ UTR of the pharmacogene- CYP2B6, was shown to be statistically significant in HepaRG cells. A recent study shows that this variant, could explain part of the interindividual variability seen in the activity of this critical pharmacogene (Burgess et al., 2017). These results demonstrate the potential of this assay to identify clinically relevant functional genetic variants.

## Discussion

We developed PASSPORT-seq to screen 100s of SNPs that are predicted to alter miRNA–mRNA interactions. The availability of pooled oligonucleotide synthesis and the NEBuilder^®^ gene assembly system have made this assay possible. Our assay builds and substantially improves the technologies that have had only limited success (Reid et al., 2009). For example, in previous high-throughput splicing assays, short inserts were underrepresented during the library construction using traditional cloning methods (Chen and Chasin, 1994; Ke et al., 2011; Soemedi et al., 2014). In contrast, our library had representation of all allele pairs. This may be explained by the cloning method we utilized, i.e., the NEBuilder^®^ gene assembly system that produces covalently closed circular plasmids as opposed to traditional cloning methods, which yield a nicked-relaxed plasmid topology. This change in topology may result in a more efficient plasmid uptake by chemically competent bacteria (Hanahan, 1983; Xie et al., 1992; Kobori and Nojima, 1993). Better transformation efficiency increases the probability of both the variant and reference plasmids being represented in the resulting pool, which is a critical prerequisite for studying the allele-specific activity of miRNAs.

The activity of miRNAs on target sequences have been studied using reporter assays where the target sites of interest are cloned into the 3′ UTR of a reporter gene followed by quantification of the reporter activity. Typical reporter assays also overexpress the miRNAs that are predicted to regulate the target site (Cloonan et al., 2008; Loya et al., 2009; Baccarini and Brown, 2010). Such overexpression, however, may not provide a physiological context to the miRNA–mRNA interaction. For example, high miRNA expression levels may force interactions with mRNAs that do not normally occur. They can also compete with the miRNA processing machinery or binding sites and alter normal miRNA function. In contrast, our assay was performed in the endogenous miRNA expression background, which provides a more physiologically relevant context of the results. In addition, we used multiple different cell lines to allow parallel testing to identify cell line-specific effects of the mirSNPs.

miRNAs regulate gene expression by either degrading the target mRNAs or by binding to mRNAs and blocking translation. Traditional luciferase assays test the effect of miRNA by measuring differences in its target protein activity. However, the differences in protein activity due to mRNA degradation and those from translational blockage will be indistinguishable using luciferase activity assays. The PASSPORT-seq assay provides additional evidence of the mechanism underlying the effect of the variant by specifically detecting only the changes in mRNA transcript levels. We demonstrated that the results obtained with our PASSPORT-seq assay did reflect those obtained with the traditional luciferase reporter assay set-up. As described above, the PASSPORT-seq assay quantifies the relative expression of luciferase mRNAs, whereas the luciferase assay measures the luciferase enzyme activity. Thus, it is not surprising that there may be differences in the magnitude of effects between the different assays. For example, the effect on the luciferase activity could be larger due to both the degradation of the mRNA and the blockade of translation. In contrast, the effect on the protein levels and activity could be smaller due to delays from the time of changes in mRNA levels until the changes in protein levels and activities are observed. Typically, changes in mRNA expression due to endogenous miRNA-mediated regulation are subtle (<30%) (Baek et al., 2008; Bartel, 2009; Denzler et al., 2016). Consequently one would expect relatively small effect sizes of the variants, which was what was seen in many of these variants. However, there are many examples demonstrating the clinical impact of these types of variants (Bhattacharya et al., 2014).

One of the key findings of these studies is the cell line-specific function of mirSNPs. This is likely in part due to the cell type specific variation in miRNA expression profiles resulting in the effect of a mirSNP being evident in one cell line and not in another (Landgraf et al., 2007; Ludwig et al., 2016). We observed that the identity and number of functional mirSNPs reproducibly varied across the different cell types. This demonstrates one of the strengths of PASSPORT-seq in that it can identify cell line-specific effects of mirSNPs. These differences were validated using a second PASSPORT-seq run that reproduced the cell line effect. Additionally, the cell line-specific effect was also observed in the application of the assay to test 111 mirSNPs in pharmacogenes. The tissue/cell specificity of mirSNP function could also explain why the effects of mirSNPs are not always consistent across studies. This further complicates the bioinformatics predictions of the functional impact of the mirSNPs. Thus, when using this assay, the cell line must be carefully chosen to reflect the cell type of interest regarding the central biological hypothesis of the study. Since studies have shown that mirSNPs affect a wide variety of biological processes such as cancer, neurodegenerative disorders, infectious diseases, cardiovascular disease, and metabolic disorders (Bhattacharya et al., 2014), the *in vitro* model for testing the mirSNPs is an important consideration.

As with any *in vitro* assay, PASSPORT-seq has some limitations. First, it detects only changes in mRNA, rather than protein levels. miRNA- mRNA interactions are known to cause mRNA destabilization, but can also lead to translational repression (Lim et al., 2005; Bartel, 2009). Since this assay does not detect changes in translation, it may underestimate the functional impact of some of the mirSNPs. Second, the variants could be affecting mRNA stability by mechanisms other than by altering miRNA targeting. For example, it could be altering RNA binding protein function that could alter the mRNA stability. Although this would need additional validation experiments to determine the mechanism of action, it would still be of biological value. Last, like other studies using the pISO plasmid, the miRNA binding site is tested in the context of the luciferase mRNA, rather than the endogenous mRNA.

In summary, the PASSPORT-seq assay is a powerful tool that bridges bioinformatic predictions and high-throughput mechanistic investigation of functional genetic variants that affect miRNA–mRNA interactions. Future efforts will be aimed toward further increasing the capacity of the assay and identifying translational effects. This assay also has the potential to be modified to be applicable to genetic variants in other functional genomic regions such as promoters and splice junctions. Collectively, these assays will be key to elucidating the mechanisms underlying the genetic contribution to the inter-individual phenotypic variability.

## Author Contributions

JI, PB, AG, and TS designed the assay. JI, KC, and PB performed the *in vitro* experiments. YH, HG, and YL performed the bioinformatic analysis. JI, KC, and TS performed the data analysis. JI, KC, YH, YL, AG, and TS wrote and/or edited the manuscript.

## Conflict of Interest Statement

The authors declare that the research was conducted in the absence of any commercial or financial relationships that could be construed as a potential conflict of interest.

## Footnotes

1 http://www.pharmaadme.org/joomla/
