# Supplementary material for: PASSPORT-seq: A Novel High-Throughput Bioassay to Functionally Test Polymorphisms in Micro-RNA Target Sites
Source: Front Genet. 2018 Jun 15;9:219. doi: 10.3389/fgene.2018.00219 (PMC6013768; doi:10.3389/fgene.2018.00219)
Supplement: Supplementary file 1 [file Table_1.PDF]

Supplementary Table 1: Table summarizing the 100 mirSNPs tested by the PASSPORT-seq assay. For each SNP, the observed percent change in the expression of the variant allele compared to the respective reference allele in predicted miRNA binding site is shown below. Blue boxes indicate a reduction in the variant allele expression and Orange boxes indicate increased expression. Dark shade of each color indicate a statistically significant change after correcting for multiple comparisons using Benjamini-Hockberg algorithm. Light shade indicated statistically significant changes obseverd before correction using the Benjamini-Hockberg algorithm. Filled in boxes indicate a result that was not statistically significant.

SNP	RS No	Reference Allele	Variant Allele	Gene	Predicted miRNA binding sites in reference allele	Predicted miRNA binding sites in variant allele	Percent-change in variant: HEK	Percent-change in variant: HepG2	Percent-change in variant: Hela
1	rs1434536	C	T	BMPR1B	hsa-miR-125b		-13.2	-11.6	-11.5
2	Rs9341070	C	T	ESR1	hsa-miR-206		1.2	5.4	0.2
3	Rs1425486	G	A	PDGFC	hsa-miR-425		-6	-7.1	6.6
4	Rs5275	T	C	PTGS2	hsa-miR-542-3p		-3.1	-4.2	3.1
5	Rs1044129	A	G	RYR3	hsa-miR-367		8.5	12.8	2.4
6	Rs16917496	C	T	SETD8	hsa-miR-502		-8.1	-2.1	5.8
7	Rs1800470	C	T	TGFB1	hsa-miR-187		1.9	0.2	5.5
8	Rs334348	A	G	TGFBR1		hsa-miR-628-5p	1.2	13.2	1.8
9	Rs8126	T	C	TNFAIP2		hsa-miR-184	-2.4	-1.3	-11.3
10	Rs28382751	C	A	XIAP	hsa-miR-542-5p		-14.7	-6	-9.8
11	Rs1799782	C	T	XRCC1		hsa-miR-138	6.2	6.7	2
12	Rs17084733	G	A	KIT	hsa-miR-221		-18.2	-24.1	-10.9
13	Rs3811463	T	C	LIN28A	hsa-let-7		3.1	5.6	22.3
14	Rs10082466	T	C	MBL2		hsa-miR-27a	2.2	-8.5	-9.1
15	Rs4245739	C	A	MDM4	hsa-miR-191		-3.5	-16.5	5
16	Rs3134615	G	T	MYCL1	hsa-miR-1827		-18.4	-21.6	-13.1
17	Rs3744483	T	C	STAT3	miR-1255a/b	miR-1739/4700-3p	-36	-5.3	-10.6
18	Rs2072081	G	T	SLC4A1	miR-3121-5p, miR-1976		19.9	18.7	15.5
19	Rs659455	T	G	GAMT	miR-1587, miR-1349/1389/378g, miR-4292	miR-4508, miR-1618/3940-3p, miR-4532	3	-2.1	24.7
20	Rs8697	A	G	UQCR11	miR-621		7.9	-11.8	-11.9
21	Rs2230567	G	A	EEF2	miR-939/1343	miR-513c/514b-5p	2.3	6.7	10.6
22	Rs8192733	G	C	CYP2A6		miR-1237/3557-5p, miR-3127-3p	-18.3	16.4	26.4
23	Rs1061609	G	C	CYP2A7	miR-4267	miR-1260/1260b/1391	0.7	-3.1	14.4
24	Rs7028	G	A	NUCB1	miR-1203	miR-4254, miR-1254/3116,	-10.3	-3.4	-22.1
25	Rs2241787	T	C	RPS5	miR-1288	miR-4731-5p	-31.8	-39.7	-17.6
26	Rs6715391	A	G	MCFD2	miR-4766-3p		0.5	3.9	-4.1
27	Rs3184780	C	T	TGOLN2		miR-3144-3p	-5.3	-9.7	-13.8
28	Rs4273214	C	A	AGXT	miR-1302/1302bd/4298		-11.9	-18.3	1.2
29	Rs745144	T	C	LBP	miR-1321/1943/4739/4756-5p		-27.3	-27.2	-28.4
30	Rs7609	A	T	NIPSNAP1	miR-577		2.5	0.7	-4.4
31	Rs16998989	G	A	DDX17		miR-1272	-0.1	-10.2	-4.4
32	Rs5875	T	A	ACAA1		miR-489/1662	-27.2	-9.3	-13.4
33	Rs12494055	T	C	CYP8B1	miR-1205		3.5	23.6	10.9
34	Rs6782601	T	C	CYP8B1	miR-587	miR-587/3692	-6.9	-6.9	-3.6
35	Rs4686848	G	A	ST6GAL1		miR-2376/3921/4653-5p	-0.8	4.1	10.5
36	Rs1059220	G	A	MRFAP1		miR-2052	17.7	2.9	27.5
37	Rs6413428	A	G	CCDC152, SEPP1	miR-4760-5p		3.8	-0.8	0.8
38	Rs60592133	T	C	HMGCS1		miR-3669	-3.4	6.2	-21.4
39	Rs698365	T	G	TMED7	miR-590-3p		2.2	-2.5	5.7
40	Rs1053411	G	C	SPARC	miR-3123		17.7	11.4	7
41	Rs112863520	C	T	HLA-A	miR-2127/4728-5p		-7.3	-14.3	-32.9
42	Rs79244404	T	C	HLA-A		miR-1304	9.2	-5.7	-14.2
43	Rs8233	G	A	TUBB	miR-508-5p/509-5p	miR-3180-5p	-11.5	-6.1	2.3

FDR Corrected Negative % Change
FDR Corrected Positive % Change
Not Sig Negative % Change
Not Sig Positive % Change
p-value (Non-FDR Corrected) Negative % Change
p-value (Non-FDR Corrected) Positive % Change

44	Rs1065711	G	A	HLA-C		miR-532-5p/511, miR-3942-3p	5.3	-3.3	-9.2
45	Rs1093	A	G	HLA-B	miR-27abc/27a-3p		7.3	10.9	1.7
46	Rs1055821	G	T	HLA-B	miR-654-5p/541, miR-4417	miR-624, miR-3147	-12.7	-39.3	-35.3
47	Rs699700	C	A	CD164		miR-3667-5p,	12.6	13	11.8
48	Rs9655959	C	T	STEAP4	miR-641/3617		-4.5	-12	-10.3
49	Rs7780066	G	A	AKR1D1		miR-3909, miR-1543/4540	3.1	15.8	5.5
50	Rs1044608	C	G	TMEM176A	miR-4274	miR-4330	-4.7	-10.6	-8.2
51	Rs3947	G	A	CTSB	miR-214/761/3619-5p		23	3.6	7.4
52	Rs17060854	C	T	SLC39A14	miR-3917		-11.8	-17.6	-8.6
53	Rs1051638	G	A	SLC39A14		miR-4520a-3p, miR-3093-3p/3138	10.3	0.4	26.1
54	Rs1051708	A	C	SLC39A14		miR-4501	0.4	-11.6	-5.3
55	Rs8218	A	C	HRSP12	miR-342-3p	miR-339b/339-5p/3586-5p, miR-10abc/10a-5p	17.4	-0.5	22.1
56	Rs4577	G	A	ALDOB		miR-3159	-0.7	4.6	3.1
57	Rs16910556	T	C	STOM		miR-25/32/92abc/363/363-3p/367, miR-376c/741-5p	12.5	19.5	10.9
58	Rs11587541	T	C	AGMAT	miR-675-5p/4466	miR-4486	38.4	82.7	67.6
59	Rs10580	A	G	C1QB	miR-3147	miR-612/1285/3187-5p	31.4	75.5	45.6
60	Rs8876	C	G	TMEM50A	miR-3672, miR-4329		-1	20.3	-1.5
61	Rs1126970	A	C	PPT1		miR-328a/328b-3p	6.5	27.8	8.1
62	Rs72897351	T	G	ECHDC2	miR-4713-5p	miR-1301/5047	-25.6	-29	-27.4
63	Rs11581122	C	T	FAM46C	miR-4292		-13.7	-20.8	-25.7
64	Rs9616	A	T	ADAR		miR-3614-3p, miR-412/3551-5p	12.4	18.4	7.3
65	Rs623479	C	T	SEC16B	miR-127-5p		10	4.8	3.2
66	Rs16828882	T	C	GLUL		miR-4713-5p	6.9	0.3	1.9
67	Rs6424898	G	A	ARPC5		miR-4461	6.6	-23.6	3.9
68	Rs4653695	A	C	EPHX1		miR-3614-5p	-36.5	-59.7	-33.3
69	Rs7770	T	C	GDI2	miR-340-5p	miR-4803	-4.7	9	3.4
70	Rs3740085	C	G	CXCL12		miR-767-5p	18.3	-0.4	18.1
71	Rs3980942	A	G	ACADSB		miR-1237/3557-5p	-18.5	-5.2	16.5
72	Rs1050040	G	A	ECHS1	miR-3928, miR-3125/3539/3916	miR-4738-3p	-1.1	-7	-0.4
73	Rs35588561	A	G	HYOU1	miR-1326/4766-5p, miR-186		7.1	29.5	4.5
74	Rs2286380	A	T	ADIPOR2	miR-758		9.1	7.8	16.6
75	Rs1803622	G	T	GAPDH		miR-3688-3p, miR-4738-3p, miR-548g	-9.8	-17.6	-13.4
76	Rs7709	C	A	C1RL		miR-1273f	21.9	19.9	-1.1
77	Rs1801153	C	T	PAH	miR-526b		2.7	-19.4	1.8
78	Rs1053948	G	C	TSC22D1	miR-4723-5p, miR-637, miR-1343	miR-1976, miR-4734	-3.8	13.8	18.5
79	Rs3094	T	C	RNASE4	miR-1302/1302bd/4298	miR-4660	-19	-37	-22.9
80	Rs1135886	G	A	ALDH6A1		miR-5096	-24.2	13.5	-4.1
81	rs8017248	G	A	ALDH6A1	miR-4787-5p		-16.6	13.9	-33.8
82	Rs11441	T	C	SERPINA10	miR-2355/2355-3p		-9.7	-8.5	-1.6
83	Rs1243165	C	T	SERPINA1	miR-296-5p, miR-361-3p	miR-2355-5p	-9.5	-9.3	12.9
84	Rs2073333	C	T	SERPINA1	miR-338/338-3p		-3.9	-14.7	-8.9

85	Rs11648372	G	A	ABAT		miR-4633-5p	10.2	8.8	5.7
86	Rs2075828	C	A	PMM2	miR-4278	miR-1587, miR-4727-3p, miR-4417	-2.4	1.4	-0.4
87	Rs6498540	A	G	PDXDC1	miR-3156-3p	miR-1260/1260b/1391	4.7	-8.2	-8.6
88	Rs1121	G	A	PDXDC1		miR-550a, miR-3064-5p/3085-3p	3.2	-9.8	20
89	Rs708274	G	T	MT1E	miR-4314		-9.6	-8.6	-9.2
90	Rs2921	A	G	IFI30		miR-214/761/3619-5p, miR-15abc/16/16abc/195/322/424/497/1907	9.6	19.4	8.3
91	Rs1045747	T	C	IFI30	miR-767-5p	miR-2116	-19.4	-39.6	-15.5
92	Rs2277706	T	G	P4HB	miR-1587, miR-4417	miR-1234	-10.7	-9.2	-12.8
93	Rs11150795	G	A	NPLOC4	miR-4417		-6.3	0.1	31.8
94	Rs7260568	G	A	GPI		miR-612/1285/3187-5p	22.2	-7.5	31.9
95	Rs696839	G	C	CYP2A7		miR-1237/3557-5p, miR-3127-3p	-16.6	2.7	-15.3
96	Rs7081	C	T	SLC5A6		miR-4535, miR-631/3661	-12	-12.3	-21.8
97	Rs4826	T	C	STARD7	miR-1236		-76.7	-86.6	-56.9
98	Rs8184	G	C	RAB17	miR-4734		3.7	-11.9	3.4
99	Rs4344931	A	C	AGXT	miR-571/4715-5p, miR-571		13.3	9	3.6
100	Rs4352283	C	A	TRIB3	miR-4523		19.8	22.7	33
