# Supplementary material for: PASSPORT-seq: A Novel High-Throughput Bioassay to Functionally Test Polymorphisms in Micro-RNA Target Sites
Source: Front Genet. 2018 Jun 15;9:219. doi: 10.3389/fgene.2018.00219 (PMC6013768; doi:10.3389/fgene.2018.00219)
Supplement: Supplementary file 2 [file Table_2.PDF]

Supplementary Table 2: Nucleotide sequences of the 5' and 3' barcoding primers used in the PASSPORT-seq assay. Unique barcode sequences are in bold characters and universal primer regions are italicized. A) List of primers used during assay development. B) List of primers used for functionally testing mirSNPs in pharmacogenes.

A.

No	Sample	5' Barcode and Primer [Forward] (27 nt)	3' Barcode and Primer [Reverse] (25 nt)
**1**	HEK Rep 1	**ATCAAC***GCCGTGTAATTCTAGGAGCTC*	**ATGCAC***CCGCCCCGACTCTAGAACG*
**2**	HEK Rep 2	**ATGTTG***GCCGTGTAATTCTAGGAGCTC*	**CAATAC***CCGCCCCGACTCTAGAACG*
**3**	HEK Rep 3	**CACAAG***GCCGTGTAATTCTAGGAGCTC*	**CATCTA***CCGCCCCGACTCTAGAACG*
**4**	HEK Rep 4	**CCAAAT***GCCGTGTAATTCTAGGAGCTC*	**CGTTTC***CCGCCCCGACTCTAGAACG*
**5**	HEK Rep 5	**CTATGG***GCCGTGTAATTCTAGGAGCTC*	**CTCCTT***CCGCCCCGACTCTAGAACG*
**6**	Hep-G2 Rep 1	**GAAACC***GCCGTGTAATTCTAGGAGCTC*	**GACAAT***CCGCCCCGACTCTAGAACG*
**7**	Hep-G2 Rep 2	**GCACTA***GCCGTGTAATTCTAGGAGCTC*	**GCGTTT***CCGCCCCGACTCTAGAACG*
**8**	Hep-G2 Rep 3	**GGTCTA***GCCGTGTAATTCTAGGAGCTC*	**GTAATC***CCGCCCCGACTCTAGAACG*
**9**	Hep-G2 Rep 4	**GTAGAG***GCCGTGTAATTCTAGGAGCTC*	**GTTAGT***CCGCCCCGACTCTAGAACG*
**10**	Hep-G2 Rep 5	**TAACCC***GCCGTGTAATTCTAGGAGCTC*	**TACAGA***CCGCCCCGACTCTAGAACG*
**11**	HeLa Rep 1	**AACGTC***GCCGTGTAATTCTAGGAGCTC*	**AAACTC***CCGCCCCGACTCTAGAACG*
**12**	HeLa Rep 2	**ACATGT***GCCGTGTAATTCTAGGAGCTC*	**ACAACC***CCGCCCCGACTCTAGAACG*
**13**	HeLa Rep 3	**ACCTTT***GCCGTGTAATTCTAGGAGCTC*	**ACGGTT***CCGCCCCGACTCTAGAACG*
**14**	HeLa Rep 4	**AGAAGG***GCCGTGTAATTCTAGGAGCTC*	**AGACGT***CCGCCCCGACTCTAGAACG*
**15**	HeLa Rep 5	**AGTGGA***GCCGTGTAATTCTAGGAGCTC*	**AGTTAC***CCGCCCCGACTCTAGAACG*
**16**	Plasmid Input 1	**TAGAAC***GCCGTGTAATTCTAGGAGCTC*	**TATGCC***CCGCCCCGACTCTAGAACG*
**17**	Plasmid Input 2	**TCAAAG***GCCGTGTAATTCTAGGAGCTC*	**TCCATA***CCGCCCCGACTCTAGAACG*
**18**	Plasmid Input 3	**TCGATT***GCCGTGTAATTCTAGGAGCTC*	**TCTACC***CCGCCCCGACTCTAGAACG*
**19**	Plasmid Input 4	**TGCTAG***GCCGTGTAATTCTAGGAGCTC*	**TGAACC***CCGCCCCGACTCTAGAACG*

B.

No	Sample	5’ Barcode *and Primer* (27 nt)	3’ Barcode *and Primer** (25 nt)
1	HeLa Rep 1	**ACATGT***GCCGTGTAATTCTAGGAGCTC*	**ACAACC***CCGCCCCGACTCTAGAACG*
2	HeLa Rep 2	**ACCTTT***GCCGTGTAATTCTAGGAGCTC*	**ACGGTT***CCGCCCCGACTCTAGAACG*
3	HeLa Rep 3	**AGAAGG***GCCGTGTAATTCTAGGAGCTC*	**AGACGT***CCGCCCCGACTCTAGAACG*
4	HeLa Rep 4	**AGTGGA***GCCGTGTAATTCTAGGAGCTC*	**AGTTAC***CCGCCCCGACTCTAGAACG*
5	HeLa Rep 5	**ATCAAC***GCCGTGTAATTCTAGGAGCTC*	**ATGCAC***CCGCCCCGACTCTAGAACG*
6	HEK Rep 1	**ATGTTG***GCCGTGTAATTCTAGGAGCTC*	**CAATAC***CCGCCCCGACTCTAGAACG*
7	HEK Rep 2	**CACAAG***GCCGTGTAATTCTAGGAGCTC*	**CATCTA***CCGCCCCGACTCTAGAACG*
8	HEK Rep 3	**CCAAAT***GCCGTGTAATTCTAGGAGCTC*	**CGTTTC***CCGCCCCGACTCTAGAACG*
9	HEK Rep 4	**CTATGG***GCCGTGTAATTCTAGGAGCTC*	**CTCCTT***CCGCCCCGACTCTAGAACG*
10	HEK Rep 5	**GAAACC***GCCGTGTAATTCTAGGAGCTC*	**GACAAT***CCGCCCCGACTCTAGAACG*
11	HepG2 Rep 1	**GCACTA***GCCGTGTAATTCTAGGAGCTC*	**GCGTTT***CCGCCCCGACTCTAGAACG*
12	HepG2 Rep 2	**GGTCTA***GCCGTGTAATTCTAGGAGCTC*	**GTAATC***CCGCCCCGACTCTAGAACG*
13	HepG2 Rep 3	**GTAGAG***GCCGTGTAATTCTAGGAGCTC*	**GTTAGT***CCGCCCCGACTCTAGAACG*
14	HepG2 Rep 4	**TAACCC***GCCGTGTAATTCTAGGAGCTC*	**TACAGA***CCGCCCCGACTCTAGAACG*
15	HepG2 Rep 5	**TAGAAC***GCCGTGTAATTCTAGGAGCTC*	**TATGCC***CCGCCCCGACTCTAGAACG*
16	HepaRG Rep 1	**TCAAAG***GCCGTGTAATTCTAGGAGCTC*	**TCCATA***CCGCCCCGACTCTAGAACG*
17	HepaRG Rep 2	**TCGATT***GCCGTGTAATTCTAGGAGCTC*	**TCTACC***CCGCCCCGACTCTAGAACG*
18	HepaRG Rep 3	**TGCTAG***GCCGTGTAATTCTAGGAGCTC*	**TGAACC***CCGCCCCGACTCTAGAACG*
19	HepaRG Rep 4	**TTCGAA***GCCGTGTAATTCTAGGAGCTC*	**TTAACG***CCGCCCCGACTCTAGAACG*
20	HepaRG Rep 5	**AAACAC***GCCGTGTAATTCTAGGAGCTC*	**TTCTGG***CCGCCCCGACTCTAGAACG*
21	Plasmid Input 1	**TAAGAC***GCCGTGTAATTCTAGGAGCTC*	**TGCTCA***CCGCCCCGACTCTAGAACG*
22	Plasmid Input 2	***TGGGAT*** *GCCGTGTAATTCTAGGAGCTC*	**TCTTAG***CCGCCCCGACTCTAGAACG*
23	Plasmid Input 3	**TCTGCT***GCCGTGTAATTCTAGGAGCTC*	**AAGAAC***CCGCCCCGACTCTAGAACG*
24	Plasmid Input 4	**AACGGT***GCCGTGTAATTCTAGGAGCTC*	**AACTTC***CCGCCCCGACTCTAGAACG*
25	Plasmid Input 5	***AATGTG*** *GCCGTGTAATTCTAGGAGCTC*	**GCAGAA***CCGCCCCGACTCTAGAACG*
