# Supplementary material for: PASSPORT-seq: A Novel High-Throughput Bioassay to Functionally Test Polymorphisms in Micro-RNA Target Sites
Source: Front Genet. 2018 Jun 15;9:219. doi: 10.3389/fgene.2018.00219 (PMC6013768; doi:10.3389/fgene.2018.00219)
Supplement: Supplementary file 3 [file Table_3.PDF]

**Supplementary Table 3: Statistical significance of differences in expression/activity of variant and reference-** a) p-values (corrected by Benjamini-Hochberg algorithm) generated by edgeR package, of the observed differences between normalized reads from variant alleles and those from the reference allele in the PASSPORT-seq assay. b) p-values from t-tests of the luciferase activity from variant and reference plasmids in the three different cell lines.
P-values <0.05 are indicated in red font.

a.

| | p-values (corrected by Benjamini-Hochberg algorithm) observed in the PASSPORT-seq Assay | | | | | | |
|---|---|---|---|---|---|---|---|
| | **Rs3134615** | **Rs10580** | **Rs4352283** | **Rs11581122** | **Rs5875** | **Rs6413428** | **Rs8184** |
| **HepG2** | **5.58E-07** | **1.63E-12** | **0.006957619** | **1.04E-07** | 0.412278483 | 0.959814218 | 0.076656796 |
| **HEK293** | **6.00E-06** | **0.004509948** | **0.003549963** | 0.052549879 | **2.59E-05** | 0.806336883 | 0.677835743 |
| **HeLa** | 0.09172685 | **1.15E-05** | **1.15E-05** | **3.30027E-05** | **0.034645158** | 0.91938467 | 0.780064143 |

b.

| | p-values observed in the Luciferase Assay | | | | | | |
|---|---|---|---|---|---|---|---|
| | **Rs3134615** | **Rs10580** | **Rs4352283** | **Rs11581122** | **Rs5875** | **Rs6413428** | **Rs8184** |
| **HepG2** | **0.013613669** | **0.004414803** | **0.019065902** | 0.221010095 | 0.492903224 | 0.415533639 | 0.060083807 |
| **HEK293** | 0.242944657 | **2.97738E-06** | **0.000516609** | 0.164958245 | **0.013292053** | 0.144723789 | 0.978267666 |
| **HeLa** | **0.001452537** | **8.57196E-05** | **0.00573863** | 0.183846408 | **0.002492274** | 0.544489105 | 0.122592006 |
