# Supplementary material for: PASSPORT-seq: A Novel High-Throughput Bioassay to Functionally Test Polymorphisms in Micro-RNA Target Sites
Source: Front Genet. 2018 Jun 15;9:219. doi: 10.3389/fgene.2018.00219 (PMC6013768; doi:10.3389/fgene.2018.00219)
Supplement: Supplementary file 4 [file Table_4.PDF]

Supplementary Table 4: Table summarizing the 111 mirSNPs in pharmacogenes tested by the PASSPORT-seq assay. For each SNP, the observed percent change in the expression of the variant allele compared to the respective reference allele in predicted miRNA binding site is shown below. Blue boxes indicate a reduction in the variant allele expression and Red boxes indicate increased expression. Dark shade of each color indicate a statistically significant change after correcting for multiple comparisons using Benjamini-Hockberg algorithm. Light shade indicated statistically significant changes obseverd before correction using the Benjamini-Hockberg algorithm. White boxes indicate a result that was not statistically significant.

RS No	Reference Allele	Variant Allele	Gene	Predicted miRNA binding sites in reference allele	Predicted miRNA binding sites in variant allele	Percent-change in variant: HEK293	Percent-change in variant: HeLa	Percent-change in variant: HepG2	Percent-change in variant: HepaRG
rs113594465	A	G	NR1I2	miR-24-3p/4257/6847-5p	miR-6075	-44.02	-48.13	-49.44	-64.43
rs12979270	A	C	CYP2B6		miR-1275/625-5p	-2.09	-1.37	-7.77	-38.99
rs17861162	C	G	CYP1A2			-25.37	-18.90	-23.70	-35.90
rs6839	A	G	SULT1A1	miR-508-5p/509-5p		-33.59	-27.65	-38.17	-34.41
rs55661878	A	G	TPMT	miR-1/206/33a-3p/613/6716-5p	miR-1292-5p/4471/8059	-17.80	-12.25	-16.69	-32.93
rs35791822	G	T	UGT2B15	miR-105/105ab		-22.88	-15.59	-22.75	-32.30
rs186026692	A	G	ABCB1	miR-4768-5p/6809-3p/6833-3p	miR-942-5p	-0.44	0.38	-4.30	-32.04
rs55839603	T	C	TPMT	miR-190a-3p/410-3p/6083	miR-379-3p/380-3p/3924/411-3p/4495	9.86	8.61	3.26	-29.37
rs1054190	C	T	NR1I2	miR-1250-5p	miR-148ab-3p/152-3p/361-5p/302ac/374a-3p/520f-3p/5094	-26.35	-10.03	-20.55	-28.84
rs3181842	C	T	CYP2B6	miR-1581/4537		-12.34	-10.73	-4.67	-28.30
rs9332242	C	G	CYP2C9	miR-143-3p/1273f/1721/4708-5p/4770/6088/8066	miR-1245b-3p/513b-3p	10.53	7.29	7.46	-28.10
rs200253092	G	A	ABCB1	miR-455-3p	miR-105-5p/607/3671/7853-5p	-4.51	-9.25	4.36	-25.86
rs189331203	C	T	SLC15A2	miR-4738-5p	miR-122-3p	-30.10	-24.95	-26.39	-25.41
rs28371763	T	A	CYP3A4			-29.74	-20.43	-27.63	-25.29
rs189039600	C	A	SLC15A2	miR-1205/17-3p/3158-5p/544a	miR-2116-5p/22-5p/4677-5p	-9.84	-24.20	-29.55	-24.59
rs150176505	C	T	ABCC2	let-7abcdefgi-5p/4458/4500/98-5p	miR-4761-5p	-24.26	-14.23	-21.60	-23.90
rs35427048	C	T	CYP1A1		miR-192-3p	7.97	-6.69	13.91	-23.83
rs55696854	G	T	DPYD	miR-1271-5p/182-5p/216b-3p/8064/96-5p		-19.21	-14.31	-26.38	-22.76
rs10211	C	T	CYP3A7		miR-4732-3p,miR-125a-5p/125b-5p/351/670/4319,miR-3920	-24.18	-6.54	-3.08	-22.37
rs3732360	C	T	NR1I2	miR-4787-3p/4763-5p	miR-501-3p/502-3p/500/502a	-9.66	-12.74	-18.19	-22.03
rs144017186	C	G	TPMT	miR-1343-5p/3150a-3p/3175/3191-5p/326/330-5p/4667-5p/4700-5p/518c-5p/637/6763-5p/7155-5p/8089/939-5p	miR-3615/6746-5p/6771-5p	-21.72	-9.96	-21.91	-21.61
rs4986884	T	C	CYP1A1	miR-665/2441/4436a	miR-1470/4497	-20.36	-5.61	-24.66	-20.64
rs71581986	T	C	SLCO1B1	miR-194-5p/802	miR-191-5p	-4.70	-3.40	-2.06	-20.64
rs2480257	A	T	CYP2E1	miR-570	miR-203	1.45	-8.36	-3.99	-20.44
rs291592	G	A	DPYD	miR-545/548p/3120-3p		-19.32	-9.62	-13.93	-19.98
rs183246640	A	G	HNF4A	miR-383-5p/4772-5p		-16.44	-19.50	-16.16	-19.75
rs4149087	T	G	SLCO1B1			5.09	3.96	14.30	-18.55
rs6103734	G	A	HNF4A	miR-345-5p/378a-5p/6838-3p	miR-1264	2.73	-0.77	-2.05	-18.30
rs10511395	C	A	NR1I2	miR-1254/3116/4254/505-5p	miR-1233-3p/1225-3p/3142/1245b-5p/6807-5p	-14.27	-13.77	-16.31	-17.66
rs1802650	A	T	TPMT	miR-186-5p/3133	miR-3613-3p/607	4.59	-7.38	-13.78	-16.38

Legend
FDR Corrected Negative % Change
p-value (Non-FDR Corrected) Negative % Change
Not Significant
p-value (Non-FDR Corrected) Positive % Change
FDR Corrected Positive % Change

rs1042389	T	C	CYP2B6	miR-4476	miR-650/650abc/3612/4266/4695-5p/765/3584-5p/4779	-1.93	0.20	-6.32	-15.58
rs76580593	G	T	NR1I2	miR-140-3p	miR-589-3p/6768-5p	-5.25	3.55	-12.05	-15.19
rs28399502	C	A	CYP2B6	miR-548q/582-3p	miR-1227	-9.10	-7.80	-11.18	-14.13
rs10460826	C	G	NR1I2	miR-4494/4499	miR-329-3p/362-3p/3941/603	3.93	3.74	5.19	-11.68
rs9457846	G	A	SLC22A1		miR-639	-9.48	-1.79	-4.17	-11.27
rs291593	C	T	DPYD			-11.84	-16.72	-13.89	-11.10
rs12107248	T	A	NR1I2		miR-29a-5p/3200-5p/3920	9.95	10.93	-0.74	-11.00
rs6438550	G	A	NR1I2	miR-1282		-0.18	6.26	-3.91	-10.49
rs149397784	C	A	NR1I2	miR-328-5p/4447/4472/4651/608/6752-5p/6756-5p/6766-5p/6795-5p/6842-5p/7109-5p/7110-5p/92a-2-5p	miR-1237-5p/1275/4488/4665-5p/4697-5p/6751-5p/6803-5p/6846-5p/6848-5p/92a-1-5p	7.37	3.99	1.44	-9.74
rs200905283	T	A	HNF4A	miR-15a-3p/4716-5p/4717-5p/5704	miR-1245b-5p/3142/4273/4312/4677-5p/6739-3p/7165-5p	1.28	-6.09	-16.71	-9.48
rs1054191	G	A	NR1I2	miR-4258/371b-3p/4707-3p	miR-515-3p/519e/550a/4769-3p/4722-3p/4763-5p	-3.20	-2.76	6.37	-9.25
rs4630	C	T	GSTT1	miR-4423-3p	miR-4503	-4.20	-0.88	-13.04	-8.83
rs12360	C	T	CYP3A7	miR-300-5p/4635/4709-3p/526b-5p/578	miR-1276/302a-5p/583/1582/4311	-8.37	-2.90	2.30	-8.71
rs17861086	G	C	CYP1A1		miR-668-3p	-8.06	-15.84	-1.37	-8.29
rs11636419	A	G	CYP1A2			8.92	-3.22	-4.11	-7.88
rs200519094	C	T	ABCB1	miR-5700	miR-369-3p/374abc-5p/5692bc/655-3p	1.47	-2.46	-5.07	-7.29
rs28969420	G	T	CYP2B6	miR-661/766	miR-4463,miR-759/2461-3p/4494	4.70	12.28	-0.54	-7.17
rs189107638	A	G	HNF4A	miR-4279/889-3p	miR-4287/4469/4685-3p/4713-5p/629-3p/6867-3p/7113-3p	9.12	3.89	9.46	-6.03
rs4986993	T	G	NAT1		miR-660	14.23	-5.93	-8.60	-5.46
rs185103906	G	T	SLC15A2	miR-188-3p/532-3p	miR-140-3p	5.66	1.37	4.90	-5.44
rs11574744	T	A	HNF4A	miR-194-3p/4654/4769-5p/34abc-5p/449abc-5p/548au-3p	miR-1233-5p/134-3p/4648/4687-3p/4654/4769-5p/6778-5p/7974	7.34	4.91	4.82	-5.09
rs183641079	C	T	CYP2B6		miR-1275/625-5p	26.01	-11.48	3.53	-3.37
rs188501488	T	A	DPYD	miR-3163/580-5p	miR-33a-3p/409-3p	11.83	8.44	4.18	-2.08
rs28364275	T	C	ABCB1	miR-302c-5p/3143	miR-514ab-3p/489-3p/3913-3p	7.07	6.48	5.73	-1.93
rs114029015	A	G	SLC15A2	miR-154-3p/34a-3p/487a-3p		16.65	9.20	13.76	-0.68
rs41282030	T	C	HNF4A	miR-539-5p	miR-3153/4668-5p/6124/6733-5p/6739-5p	4.87	4.33	-2.44	-0.48
rs114764820	T	C	HNF4A	miR-3691-3p/2114-5p	miR-3090/4726-3p/500b-3p	13.21	13.51	7.86	0.03
rs138053056	C	T	CYP2B6	miR-744-5p		8.68	10.61	21.02	0.43
rs28364280	G	A	ABCB1	miR-138-1-3p/3682-5p	miR-374ab-5p/5003-3p	-9.24	-5.65	-16.40	0.95
rs8330	G	C	UGT1A		miR-1286/2441/4436a	5.44	15.67	4.66	1.33
rs1042157	C	T	SULT1A1	miR-4532/4449	miR-1915/3090/4726-3p	8.17	17.29	23.79	1.49

rs41280258	G	A	HNF4A	miR-1291/1291ab/328a-3p/328b-3p/938/663b/6775-3p/6851-3p/7108-5p		12.22	29.70	-4.64	2.13
rs56307258	C	A	CYP2E1	miR-510-3p/570-3p	miR-548a,ar,az,e,f-3p/5582-3p/590-3p	15.69	11.87	2.94	2.30
rs2151562	T	C	DPYD		miR-3606/3591-5p/3174/921	12.72	-1.19	5.10	2.41
rs11574745	C	T	HNF4A	miR-493-3p	miR-4699-5p	17.32	0.70	8.12	2.97
rs200737867	G	A	NR1I2	miR-3615/3917	miR-2682-3p/3689d/432-5p/4443/6515-5p/6781-3p/6851-5p	-5.00	-9.06	13.09	4.07
rs3814058	T	C	NR1I2	miR-489-3p/1662/514ab-3p	miR-1256/182-5p/96-5p/507/1271-5p/4642	14.55	12.02	9.42	4.52
rs201524771	A	C	ABCB1		miR-224-3p/30abcde-5p/522-3p	9.88	-6.54	9.00	4.58
rs79775553	C	G	SLCO1B1	miR-6740-5p	miR-9-5p	7.21	5.68	6.89	5.00
rs180751034	G	T	SLC15A2	miR-20a-3p/217/6807-3p	miR-183-3p/4680-3p	-12.31	-4.89	-8.55	5.41
rs139849737	T	C	CYP3A7	miR-3919/4786-3p	miR-505-5p/2467-3p/6487-5p	12.67	8.88	6.27	7.75
rs3212210	A	C	HNF4A			23.52	4.25	-2.93	8.15
rs201925035	A	C	GSTP1	miR-5680/590-3p	miR-561-3p	19.35	23.03	15.27	9.31
rs8192733	G	C	CYP2A6	miR-1581/4537	miR-1233/1237/3557-5p/3127-3p/1225-3p	37.95	21.82	29.43	10.07
rs184161277	T	C	DPYD		miR-223-5p	8.73	6.97	12.28	10.15
rs3732358	G	A	NR1I2	miR-500b-3p/597-5p/6814-5p/6879-3p		21.25	9.51	13.22	11.11
rs2472683	A	G	NR1I2	miR-1292/4471/3189-3p	miR-3909	25.09	9.50	11.76	11.13
rs41285690	A	G	DPYD	miR-1279/5007-3p	miR-4724-5p/485-3p/539-3p	32.43	17.18	12.94	11.21
rs707265	A	G	CYP2B6		miR-1623/4269/3622a-5p	11.50	16.39	14.04	11.24
rs185946310	C	T	NR1I2	let-7abcdefgi-5p/3908/3942-5p/4458/4500/4703-5p/4766-3p/98-5p		7.33	0.40	-10.00	11.68
rs79132805	G	A	SLCO1B3	miR-4436b-5p		-8.50	6.96	4.62	11.88
rs3732359	G	A	NR1I2	miR-2284s/3136-3p/362-5p/500b	miR-501-5p/362-5p/500b	1.60	-9.90	0.67	11.91
rs183800671	C	G	CYP3A7		miR-219a-5p/4782-3p/6766-3p	19.82	8.21	17.45	12.46
rs1142365	A	G	TPMT	miR-411-5p	miR-199ab-3p/222-5p/30ade-3p/3129-5p/936	18.65	3.07	12.77	13.18
rs15524	T	C	CYP3A5	miR-500a		22.34	20.26	30.60	13.83
rs56160474	T	C	DPYD	miR-641/3617-5p	miR-4717-3p/4524a-3p	27.77	20.43	22.43	14.84
rs146050194	C	T	CYP2B6	miR-1275/625-5p		23.74	4.11	17.86	15.73
rs71581985	T	G	SLCO1B1	miR-106a-3p	miR-4659ab-5p	27.67	2.63	9.87	17.59
rs9465100	G	A	TPMT	miR-10ab-5p/339-5p/4725-5p/4786-5p/504-5p/769-5p	miR-4421/5699-3p/6748-3p	11.22	20.22	14.01	18.90
rs4149088	A	G	SLCO1B1			21.74	8.44	11.60	19.02
rs17470762	T	C	DPYD	miR-1277-5p/5011-5p/889-3p		8.06	6.35	1.52	19.28
rs1042640	G	C	UGT1A		miR-885-3p	20.98	10.17	27.97	19.83
rs4803420	G	T	CYP2B6			-17.02	-18.32	-27.85	21.08
rs1038376	A	T	CYP2B6		miR-1588/3914	16.64	16.44	29.59	22.00
rs189810658	C	G	TPMT	miR-411-5p/6832-5p	miR-2681-5p/28-3p	21.20	15.38	24.31	23.80
rs112532686	T	C	SLC15A2		miR-145-5p/3180-5p/5195-3p	8.74	20.43	18.20	26.23
rs13860	T	C	SLC15A2	miR-203a/4766-5p	miR-1273f/143-3p/4251/4303/4329/4708-5p/4770/6088/6761-5p	25.06	39.91	45.86	26.42

rs55818790	A	G	TPMT	miR-33ab-5p/486	miR-20a-3p/217/501-3p/502-3p/6807-3p	**16.47**	**14.85**	**13.88**	**27.86**
rs3814057	A	C	NR1I2	miR-148b-5p/3168/2116/6800-5p/6802-5p/6874-3p		**20.21**	**11.25**	**21.94**	**29.95**
rs17161788	A	G	CYP3A5	miR-3973	miR-330-3p	**8.62**	**7.59**	**10.82**	**31.58**
rs10929303	T	C	UGT1A			**10.15**	**6.99**	**8.08**	**32.67**
rs202026600	T	A	ABCB1	miR-485-3p/539-3p	miR-582-5p/600	**9.02**	**11.05**	**24.60**	**34.06**
rs7260525	A	G	CYP2B6			**10.23**	**46.59**	**34.02**	**36.72**
rs1058932	C	T	CYP2C8		miR-214/761/3619-5p/922/1704/4291	**12.83**	**-0.47**	**5.67**	**40.22**
rs2480256	A	G	CYP2E1	miR-570		**46.78**	**30.68**	**35.33**	**42.53**
rs7246465	T	C	CYP2B6		miR-548ad/337-5p	**14.07**	**7.68**	**17.32**	**45.26**
rs12721615	A	G	NR1I2	miR-758-3p		**12.67**	**7.78**	**16.73**	**46.82**
rs45539742	C	G	NAT2	miR-15a-3p/1972/4529-5p	miR-122-3p	**38.38**	**17.16**	**17.05**	**48.67**
rs139156785	G	A	CYP1A1	miR-1236-3p/204-5p/211-5p/4755-5p/5006-3p/5008-3p/623	miR-412-3p/4753-3p/6515-3p/6754-3p/6809-3p/6837-3p/942-5p	**33.59**	**27.99**	**7.86**	**52.14**
rs7081484	C	T	CYP2E1		miR-4450/4525/5010-5p/506-5p	**35.99**	**11.86**	**30.16**	**64.56**
rs11086926	T	G	HNF4A		miR-4655-5p/4508	**91.29**	**65.82**	**75.25**	**144.76**
