# Supplementary material for: PASSPORT-seq: A Novel High-Throughput Bioassay to Functionally Test Polymorphisms in Micro-RNA Target Sites
Source: Front Genet. 2018 Jun 15;9:219. doi: 10.3389/fgene.2018.00219 (PMC6013768; doi:10.3389/fgene.2018.00219)
Supplement: Supplementary file 5 [file Supplementary_File_1.pdf]

Supplementary File 1: EdgeR script for performing differential expression analysis

```
args <- commandArgs(trailingOnly = TRUE)

cat("args:\n"); print(args)

if(length(args) != 2) {

  cat("Wrong input argument\n");

} else if (file.exists(args[[1]])) {

  cat("Input file not exist or inaccessible.\n");

  if(!grepl(".RDS$", args[[1]])){

    cat("Unexpected input file format, RDS expected.\n")

  }

}else {

  count_fnm <- args[[1]]; ## input count matrix

  output_fnm <- args[[2]]; ## output file name

}

## DE analysis library

library(edgeR);

counts.df <- readRDS(file = count_fnm);

## Note: the data columns in count.df should be consistent with

##      "bar_code", "genotype", "run" specified here.

bar_code <- rep(rep(paste0("rep_", c(1:5)), each = 2), 2);

genotype = factor(rep(c("WT", "SNP"),10),levels=c("WT","SNP"));

run = c(rep('fstRun',10),rep('sndRun',10));

design <- model.matrix(~bar_code+run+genotype);

y = DGEList(counts=counts.df);

lib.size <- y$samples$lib.size[seq(1,ncol(y$counts)-1, by=2)] + y$samples$lib.size[seq(2,ncol(y$counts),
by=2)];

lib.size <- rep(lib.size, each=2);

y$samples$lib.size <- lib.size;

rownames(design) <- colnames(y);
```

```
y <- estimateGLMCommonDisp(y, design, verbose=TRUE);

y <- estimateGLMTrendedDisp(y, design);

y <- estimateGLMTagwiseDisp(y, design);

fit <- glmFit(y, design);

lrt <- glmLRT(fit);

result <- topTags(lrt,n=nrow(x),sort.by="none")$table;

write.csv(result, file = output_fnm, row.names = F);
```
